# A retrospective audit of general practitioner’s referrals to Guys and St Thomas’ specialist menopause clinic between 2021 and 2022

**DOI:** 10.1177/20533691241239485

**Published:** 2024-03-21

**Authors:** Jocelyne Tedajo Tsambou, Deborah Bruce, Debra Holloway, Janice Rymer

**Affiliations:** 1 Obstetrics and Gynaecology Department, 2112 Guy’s and St Thomas’s GP Locum, Bedfordshire, UK; 2 Department of Obstetrics and Gynaecology, Guy’s and St Thomas’s, McNair Centre - Women's Services, London, UK; 3 School of Medicine, 8945 University of Surrey, Guildford, UK; 4 Obstetrics and Gynaecology and Dean of Student Affairs, King’s College London, London, UK

**Keywords:** Alternative therapies, breast cancer, menopause, hormone replacement therapy, urogenital atrophy

## Abstract

**Purpose**: We performed a retrospective audit of General Practitioners’ (GPs) referrals to the specialist Menopause Clinic at Guys and St Thomas’s (GSTT) between 2021 and 2022. We aim to establish the indication for the referrals and whether they were compliant with the National Institute for Health and Care Excellence Guidance NICE.

**Background**: GSTT is a teaching hospital in central London that educates gynaecologists in training as well as (GP) for specialist certification in Menopause. The menopause clinic receives approximately 580 GP referrals per month from South East London practices. The current waiting time for an initial appointment is up to 1 year. This delay reflects an increase in demand for menopause care and a deficit in service provision in many areas of the UK.

NICE has recommended that GPs refer complicated cases to menopause specialists, with 11 specific criteria.

**Study Sample and Data Collection**: We randomly selected 50 patients referred to the GSTT clinic by a GP between 2021 and 2022. Patient data were collected, including patient demographics, date of referral, indication for referral, date of consultation, waiting time, past medical history, investigations, and treatment instigated during the appointment.

**Results**: The majority of referrals to the GSTT menopause Specialist clinic met the NICE guidelines (76%). One-sixth of the referrals could have been prevented or managed through alternative routes. Finally, although this is a small study, some patient unmet needs (PUNS) and GPs’ educational needs have been identified.

## Introduction

The average life expectancy for women in the UK is 82.3 years, and with the average age of menopause being around 51 years, women can expect to live a third of their lives post-menopause.^1,2^ This demographic shift, coupled with the increasing awareness and changing societal attitudes towards menopause, has led to an anticipated increase in demand for menopause care in primary care and a subsequent rise in referrals to secondary care. The increased awareness of menopause care attributed to the Channel 4 documentary ‘Sex, Myths and Menopause’, (‘The Davina effect’) along with the efforts of campaigners, have also played a significant role in fuelling this demand.^3^

However, the lack of specialist services^4,5^ poses a significant challenge in meeting this increased demand. The current state of menopause care in the UK is characterized by a lack of specialist services, which can be attributed to several factors. Inadequate training and confidence among primary care providers,^6^ workforce shortages, geographical challenges, the Covid-19 pandemic^1^ budgetary constraints, and lack of prioritization by the UK Department of Health and Social Care, in menopause care have contributed to the insufficient support for menopausal women.

To address these barriers and ensure comprehensive and high-quality menopause care across the UK, the British Menopause Society (BMS) has put forth a vision for menopause care, emphasizing the need for local authorities to review and redesign their service provision, clarify General Practitioner (GP) referral pathways, and identify appropriate referrals.^1^ Additionally, the National Institute for Health and Care Excellence (NICE) has recommended that General Practitioners (GPs) refer complicated cases to menopause specialists, with 11 specific criteria (Table 1 Blue column) outlined in their guidelines (NICE NG23, 2015).^7^ The Department of Health and Social Care (DoHS) and the newly established Menopause Taskforce have recognized the need to support menopause services and care to address the gender gap in healthcare in 2022.^8,9^

**Table 1.** Nice guidelines NG23 (criteria for seeking further specialist menopause advice) and GP referral in each category.

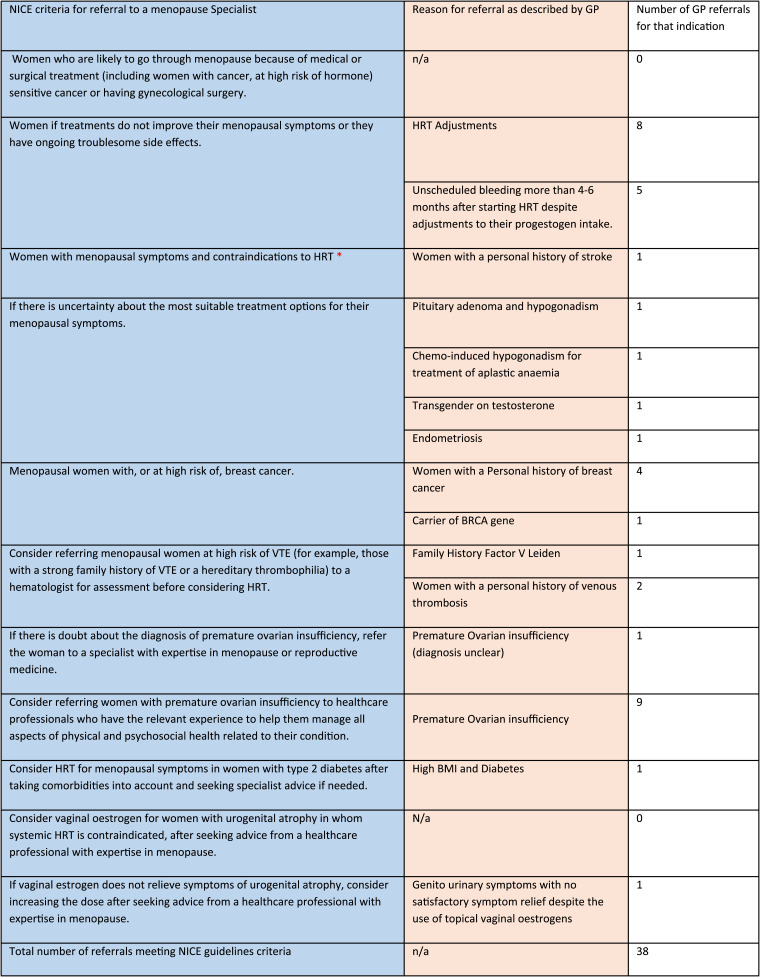

## Background

Guys and St Thomas’ (GSTT) is an NHS (National Health Service) Foundation Trust in central London. It is a well-established tertiary referral centre and a teaching hospital training Gynaecologists, Nurses, and General Practitioners for specialist certification in Menopause.^10,11^

The Menopause clinic receives referrals from South-East London GP Practices. On average it received 580 GP referrals per month between March 2022 and 2023 and an average of 650 referrals per month including tertiary referrals. Each referral is triaged and either booked into a routine appointment, or advice and guidance is given. The current waiting time for an initial appointment is up to 1 year. Several reasons explain this delay. The clinic relied on a limited number of four Gynaecologist Menopause specialists including a retiring Nurse Consultant Specialist and only run every 2 weeks. This delay reflects an increase in demand for menopause care and a deficit in service provision in many areas of the UK. This problem was exacerbated by the service disruption caused by the 2020 Covid-19 pandemic.

## Aim

This is a retrospective audit, performed between 2021 and 2022, aimed to establish the reasons for GP referrals to GSTT Menopause Clinic and whether the referrals meet the NICE guidance NG23^7^ updated in 2019. This study aims to offer guidance on adapting menopause services to optimize systems and reduce pressure in secondary care. Additionally, this work could be instrumental in enhancing the quality and efficiency of menopause services.

## Method

In this audit, we randomly selected 50 new patients referred to the GSTT Menopause Clinic between 2021 and 2022 for the management of menopause. Primary Patient data were collected, including patient demographics, date of referral, indication for referral, date of consultation, past medical history, investigations, and treatment instigated during the appointment.

## Results

**Figure 1.**
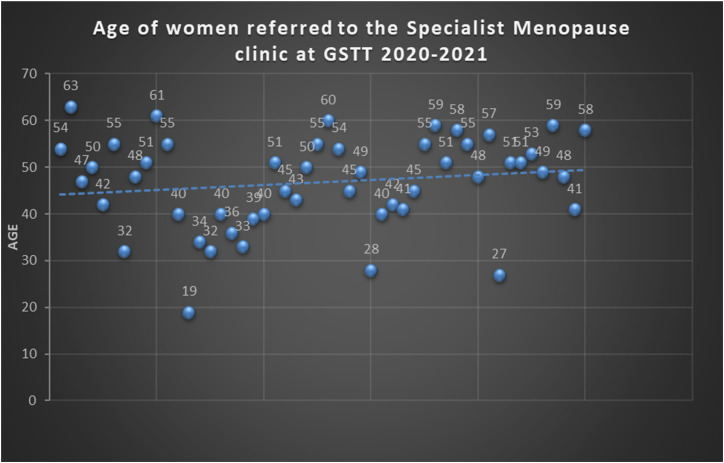
Age at the time of the referral to GSTT menopause clinic NICE guidelines NG23 (criteria for referral to a menopause specialist).

Women referred to the GSTT Menopause clinic were aged between 19 and 63 years old (Figure 1). The mean age for referral was 46 years old. The delay between the day of the referral and the time the patient was seen was, on average, 4 months ranging between 2 and 16 months.

In this audit, 76% percent of GP referrals met the NICE guidelines NG23 for a referral to the Menopause specialist clinic (Table 1). However, 24% (12 cases) were not compliant (Table 2). Overall, 76 % of referrals met the Nice Guidance (NG23) and were as follows:

 • 26% of referrals were for women who did not improve with their existing Hormone Replacement Therapy (HRT) or had ongoing troublesome side effects.
Unscheduled bleeding represented the most common and serious side effect that needed further investigation (one-third in this category).
 • The second most common referral was for women with Primary Ovarian insufficiency (POI) (20%). Most women had an established diagnosis of POI and were referred for their long-term management. Only one patient had an equivocal diagnosis and was referred for further assessment.
 • Menopausal women with, or at substantial risk of, breast cancer represented 10% of the referrals. One patient is a carrier of the BRCA1 gene.
 • Menopausal women at elevated risk of venous thromboembolism (VTE), for example, those with a strong family history or a hereditary thrombophilia were referred directly to the menopause clinic rather than the haematologist for an assessment. One woman had Factor V Leiden and 2 women had a personal history of DVT. This category represents only 0.06 % of our referrals.
 • A few complex cases were also referred for an opinion as the GP was unsure about the most suitable treatment options for the patient’s menopausal symptoms. Cases included patients with endometriosis, pituitary adenoma and hypogonadism, chemotherapy-induced hypogonadism for treatment of aplastic anaemia, and transgender on testosterone.
 • Finally, advice was also sought for a patient with diabetes and associated hypertension and for a patient with genito urinary symptoms not improved with usual treatment. This represented a small number (one in each category).

**Table 2.** GP referrals did not meet the nice guidelines (NG23).

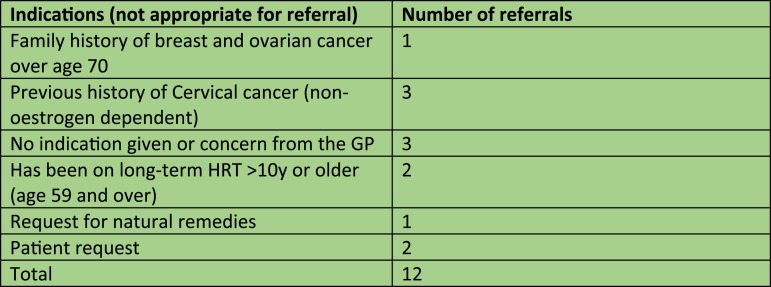

### GP referral not meeting nice guidelines NG23 (2015)

24% of patients were referred for reasons that did not meet the Nice Guidelines NG23 (2015) standard for referral.
 • Common referrals included patients with a previous history of non-estrogen-dependent cancer. As such 3 women were referred with a previous history of cervical cancer.
 • Referrals were also made over age concerns. A GP referred a patient as she was 59 years of age and wished to start HRT. Another referral was made as the GP felt that it was necessary to stop HRT as the patient had been taking it for over 10 years.
 • Referrals due to family history and associated genetic concerns also represented less than 1%. In this study, one patient was referred with a family history of Factor V Leiden another was because of a family history of breast cancer and ovarian cancer.
 • Other Referrals: 3 patients were referred with no concerns from the GP. Two patients had requested to see a specialist and another one was referred because she was interested in a more holistic approach to menopause and chose to use natural remedies to treat her condition.

## Discussion

### Regarding inappropriate referrals

#### Gynaecological cancer

Referrals were made for women with a personal history of gynaecological cancer; in this study, three women were referred with a previous history of cervical cancer. Most cervical cancers are squamous cell carcinoma so they are non-estrogen dependent.^12^ A systematic review conducted in 2021 of the PubMed English literature determined HRT had no detrimental effect on cervical cancer oncological outcomes, while several benefits were demonstrated, including reduced metabolic risk and improved quality of life, suggesting HRT should be offered to young cervical cancer survivors for early menopause management.^12,13^ Providing clear guidance to GPs and GP champions in menopause for managing menopausal women with a personal history of gynaecological cancer through training and conferences would help reduce the delay in treating affected women.

#### HRT and age concerns

Whilst it is true that when started within 10 years of menopausal symptoms or before age 60, HRT can provide cardiovascular protection, there is no proven evidence of increased cardiovascular events in women who start HRT even 10 years after the menopause as evidenced by the Cochrane research and the long-term follow-up data of the WHI (BMS, 2020).^14^ However, it is important to be aware of the recommendation to start a lower dose and in a transdermal form^14^ as it carries a lower risk of VTE and Stroke.

Similarly, a GP sought assistance to stop HRT for a patient who had been using HRT for over 10 years. The GP tried to stop HRT gradually, but the patient’s symptoms returned, adversely impacting her quality of life. The GP was concerned about the long-term risks of HRT. However, as Dr John Stevenson commented on behalf of Women’s health concerns the BMS ‘*use HRT at the lowest dose for the shortest time*” *was never evidence-based, and there is now evidence against it. The other mantra that HRT must be stopped on safety grounds after 5 years duration should be blown out of the water*!’. As such it is important not to set arbitrary limits on the duration of HRT use. If the patient has suffered ongoing symptoms the risks are usually outweighed by the benefits.^14^

A decision should be made on an individual basis and with the informed consent of the patient. In both cases, the GP concerns could have been addressed using an advice and guidance service (via a telephone helpline or email) and by – educating GPs regarding the new research findings.

##### Genetic concerns

Although one could appreciate the GP’s apprehension, consulting patient decision aids (PDAs) provided by NICE can help a risk assessment and may have prevented a referral to secondary care. NICE clinical guidance CG164 is a reliable source of information and advice.^15^

##### HRT and natural remedies

As a General Practitioner, we do not possess a comprehensive knowledge of complementary medicine and neither do consultant gynaecologists specialized in menopause. However, The International Menopause Society IMPART online learning for healthcare professionals may help close the gap.^16^ Directing patients to a GP with a specialist interest in menopause and with the support of the complementary medicine society expert (CMA) could offer more answers than a referral to the Menopause Specialist clinic. The CMA has a ‘woman health section’ with some advice regarding the brain and maintaining cognitive functions, acupuncture, and vasomotor symptoms.^17^

##### Other referrals

Three patients were referred with no concerns from the GP, but at the patient’s request reflecting either a lack of confidence in the GP to manage the menopause or a lack of trust from the patient in the primary care service to treat their symptoms effectively. Again, an advice line could have supported the GP to look after these patients.

## Conclusion

Most referrals to the GSTT Menopause Specialist clinic met the NICE guidelines (76%). Few referrals could have been avoided or dealt with by using other pathways such as advice and guidance services (via email or telephone helpline) where available, the BMS forum that provides guidance to GPs. In addition, the current NICE guidance also refines the criteria for the referral to the specialist menopause clinic.

Finally, although this is a small study, some patient unmet needs (PUNS) and GP’s educational needs have been identified and need to be addressed through conferences and webinars. In my view, the lack of GP confidence stems from several factors including the fact that menopause research is lacking; there is only sparse GP training in this subject and the existence of old confusing HERS and WHI trials. It goes without saying menopause treatment is complex and the management encompasses several specialties.

While we recognize a gap in training amongst GPs, given the current pressure on Primary Care Services, we believe it is more realistic to suggest that each locality or PCN (Primary Care Network) should encourage and invest in GP menopause specialists so that they can provide triage, timely advice, and care in response to the growing demand. NICE NG23 also suggests the setup of a regional menopause specialist’s clinic to mitigate the waiting list.

Hacking 2022 suggests the commissioning of well-spread local community diagnostic centres (CDCs) throughout the country to significantly enhance access to care for a larger number of women, addressing a key concern highlighted by the UK Menopause Taskforce.

The South East London network at GSTT is working to develop up-to-date guidance for GPs revise the referral criteria, organize group consultations for patients to reduce the waiting list, and provide training for GPs. The British Menopause Society (BMS) on the other hand is undergoing changes in its education program starting in 2024, leading to the discontinuation of the BMS Postgraduate Programme in Menopause Care (BMS PPMC) courses and instead providing a complete online course that will lead to the obtention of the equivalent of the BMS Certificate in the Principles and Practice of Menopause Care without the need to secure a BMS menopause trainer.

NHSE/I have developed an Optimal Pathway for GPs which is currently being piloted in the Midlands. The pathway is designed to assist GPs in delivering menopause care and will be a great resource for GPs who are not menopause specialists.
